# Using Imagery Rescripting as an Early Intervention for Depression in Young People

**DOI:** 10.3389/fpsyt.2021.651115

**Published:** 2021-08-23

**Authors:** Victoria Pile, Patrick Smith, Jennifer Y. F. Lau

**Affiliations:** ^1^Department of Psychology, Institute of Psychiatry, Psychology and Neuroscience, King's College London, London, United Kingdom; ^2^Department of Psychology, Royal Holloway, University of London, Egham, United Kingdom

**Keywords:** depression, adolescence, early intervention, imagery rescripting, mental imagery

## Abstract

Innovation is urgently needed for school-based early interventions for depression. Imagery rescripting for aversive memories has been shown to be a valuable therapeutic approach in adults. Yet it is rarely applied to young people or to depression. This is surprising given that intrusive images of aversive memories are implicated in the development and maintenance of depression. We review the literature and describe the co-development of an imagery rescripting protocol for young people (age 16–18) with high symptoms of depression. To contextualize and illustrate this approach, we identify three themes of negative images emerging from the 37 participants who completed imagery rescripting and provide a detailed case example for each theme. The identified themes are *failure, interpersonal adversity*, and *family conflict or disruption*. Given that there is some therapist concern about using imagery rescripting, we highlight any reported negative consequences of engaging in imagery rescripting. We propose that imagery rescripting is an acceptable and potentially effective tool for early intervention in depression, which is significantly underutilized in current practice.

## Introduction

Impairing symptoms of depression are present in 5% of young people (YP) aged 17–19 (1). However, ~75% of YP with depression do not receive an intervention (2) and current evidence-based psychotherapies for YP show little advantage over usual care, if any (3, 4). Novel early interventions are needed, particularly those that target cognitive mechanisms known to drive and maintain depression (5, 6). Traditionally, interventions for adolescent depression focus on verbal restructuring of negative thoughts and/or behavioral activation. Intrusive memories are a key maintaining factor for depression and imagery rescripting (IR) is a psychological technique aiming to reduce distress caused by aversive memories. IR is a highly effective transdiagnostic technique in adults and is likely to be developmentally appropriate. For example, young people rely more heavily on image-base processing (relative to adults) and neurocognitive development during adolescence may impact on vulnerability to distressing mental imagery (7). More generally, adolescence is a period characterized by increased flexibility and learning potential (8), and targeting mental imagery in interventions could have long-lasting benefits (9).

IMAGINE (Integrating Memories and Generating New Experiences) is a novel intervention aiming to reduce depression in YP aged 16–18. It combines IR with techniques to enhance positive future images and autobiographical memory specificity (10, 11). IMAGINE consists of four, 90 minute sessions delivered face-to-face in schools. The intervention is manualised and accompanied by a workbook. It has been initially tested in a case series (12) and feasibility RCT (13, 14) with promising results. Here, we focus on using IR with YP. First, we review the literature. Second, we describe the co-design of the IR protocol and developmental adaptations. Third, we review the types of images reported by 37 participants, identifying key themes, each with a case example. This aims to identify the types of images amenable to IR, presenting in the context of adolescent depression. Finally, we report any negative consequences (e.g., risk issues, decline in mood) from IR. This is important as IR is rarely used in current practice, perhaps due to fears associated with worsening symptoms.

## Current Literature

A meta-analysis of 19 trials using IR in adults across different disorders (15) demonstrated large effects of IR in reducing symptoms from pre-treatment to post-treatment and at follow-up (Hedges' *g* = 1.22 and 1.79, respectively). In young adults (age 18–24), two RCTs (16, 17) have examined IR to reduce social anxiety. They showed large within group effect sizes (*d* = 2.10; *d* = 1.09; *d* = 3.00), and large effects compared to passive [*d* = 0.83; (17)] and active control groups [*d* = 1.02; (16)]. IR has also been applied to other types of memories, for example a case series (18) (*n* = 9) indicated that two-sessions of IR could reduce test anxiety in university students. Excluding our work, only 2 single case studies (19, 20) have used IR in YP under age 18; these showed promising results in reducing anxiety.

Some studies have examined the mechanism of action, with suggestions that IR reduces distressing negative images through reducing avoidance, enabling emotional processing, and/or updating the meaning/content of the image. Studies with unselected samples (in the 18–24 age range) have illustrated that IR can update the meaning and reduce the distress and anxiety associated with an aversive memory (21, 22). Whilst requiring replication, there is some experimental evidence (23) to support suggestions that IR may be less distressing than techniques using exposure and preferable to positive imagery alone, which may represent another form of avoidance (24).

## Protocol Development

**Figure 1:**
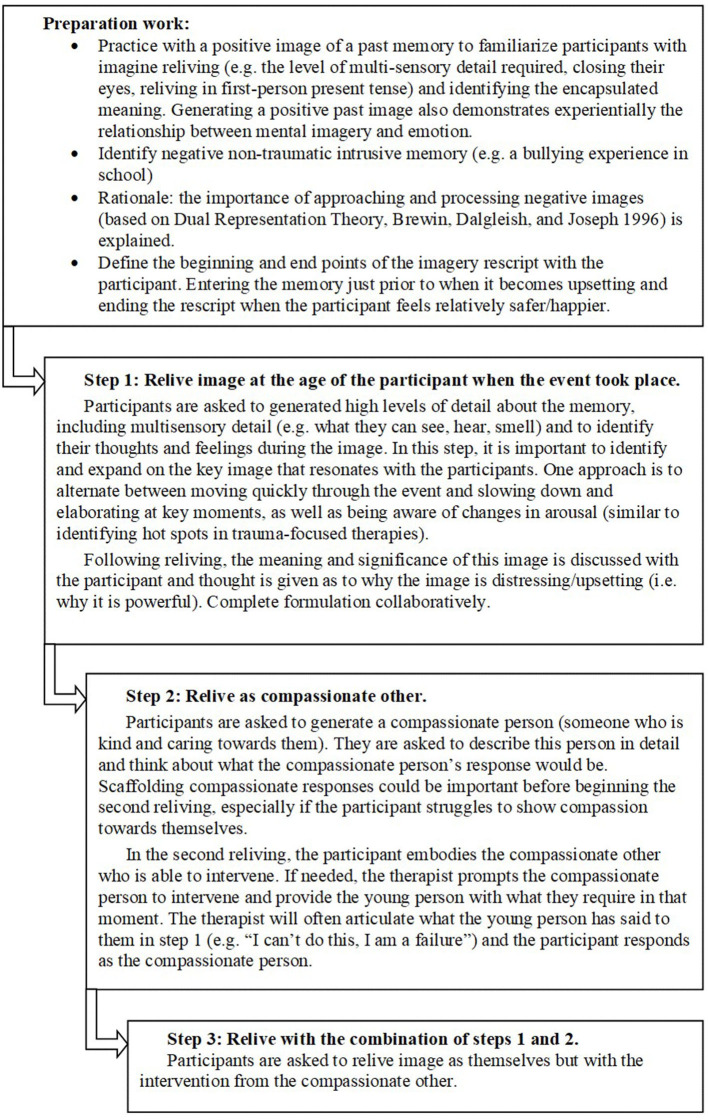
Protocol for imagery rescripting.

IR follows three steps, recalling the image in a different way in each step (see Figure 1). The protocol was co-designed with adolescents based on previous adult literature (11, 25). IR took place in a single 90-min session, with preparation work completed in the previous session. Co-design included consulting YP and adults with lived experience, parents of YP with lived experience, teachers, and clinicians. Overall, more than 60 people with lived experience were involved. This includes two service user consultants who provided consistent oversight throughout the project. Consultation and developmental adaptations have included:

- Discussions informing early development of ideas with YP with lived experience and their parents (attending Child and Adolescent Mental Health Services) and a workshop run by a national YP's mental health charity. This highlighted having the inclusion criteria as YP with symptoms of depression (rather than diagnosis, this also reflects current practice in UK services) and delivering the intervention in schools (rather than NHS clinics).
- Individual feedback on intervention content during discussions in clinical and research settings with YP and adults with lived experience, parents, and clinicians. Feedback highlighted that targeting distressing negative memories was acceptable and therapeutically valuable. Adaptations to aid engagement in IR included, firstly, psychoeducation on the impact that memories have on our mood, behavior and sense of self and how this interacts with the meaning that we ascribe to memories; secondly, an extensive practice with a positive image to familiarize YP with imaginal reliving (e.g., the level of multi-sensory detail required).
- Advisory groups gave feedback on rationale and methodology. The Biomedical Research Centre Service User Advisory Group (SURE, King's College London) provided feedback and the project was presented at three meetings with the Young Person's Mental Health Advisory Group. This included reviewing summaries, methods and ethical considerations and recommendations for enhancing recruitment. Whilst they emphasized the importance of school-delivery, adaptations for this were discussed. For example, the therapy space may feel less safe than a clinical setting and the memory is likely to be more recent for a YP (including reminders being more likely). Discussions identified the importance of rescripting a non-traumatic image, to take regular mood ratings, and to actively encourage YP to be compassionate toward themselves (e.g., having time to process the session and not to return straight to class).
- Discussion with teachers on current practice for pupils with depression and implementation feasibility. This highlighted considerations for intervention length and practicalities around school-delivery.

### Context: Imagine Methodology

IMAGINE was developed with inner-city schools with high diversity. In the participating schools, on average, 29.2% of the pupils were eligible for free school meals (UK average 12.4%) and 59.9% of pupil's first language was not English (UK average 16.6%). Thirty-eight participants were enrolled to complete IMAGINE across the case series and RCT (age $\bar{x}$ = 17.06, *SD* = 0.56; 63% female; 74% Black, Asian, and Minority Ethnic individuals). One participant did not begin and one participant discontinued the intervention. The feasibility randomized controlled trial (RCT) was prospectively registered (https://www.isrctn.com/; ISRCTN85369879). Ethical approval was obtained from the Psychiatry, Nursing and Midwifery Research Ethics Committee at Kings College London (ref: HR-16/17-3548). All participants provided written and informed consent. Inclusion criteria were: aged 16–18; being able to provide informed consent; being willing to engage in psychological therapy and complete assessments; and scoring above cut-off for depression (score of 20) on the Mood and Feelings questionnaire [MFQ; (26)] at two time points. Exclusion criteria were: diagnosis of intellectual disability or significant head injury, neurological disorder or epilepsy; unable to fluently communicate in spoken English; unable to give informed consent; factors contra-indicating imagery rescripting (e.g., high levels of current risk); currently receiving another psychological intervention (including school counseling); experiencing distressing psychotic symptoms or depressed in the postnatal period (participants with comorbid physical illness or non-psychotic disorders, such as anxiety, were not excluded). A clinical interview was completed at first interview to check inclusion/exclusion criteria, assess risk, and previous diagnoses. Four participants reported mental health diagnoses (2 major depressive disorder, 1 bulimia nervosa, 1 post-traumatic stress disorder) and one a diagnosis of autism. Eight participants reported other diagnoses including asthma (*n* = 5); learning difficulties (*n* = 1); Turner syndrome (*n* = 1); irritable bowel syndrome (*n* = 1).

There were three assessment timepoints (pre-intervention, post intervention, and 3-month follow-up) with depression measured using the MFQ. The therapist completed an individual case report form each session, including a description of the session and a summary of the generated images. Please see trial protocol for full methodology (13).

## Themes of Negative Images

Thematic analysis identified three themes (from the 37 images). These were *failure, interpersonal adversity*, and *family conflict or disruption*. First, two researchers familiarized themselves with the descriptions of the negative images and generated themes. Themes were reviewed and then images independently categorized with high agreement (95%). One image was categorized separately as representing a personal health issue (although there was overlap with the failure theme as key cognitions included “I'll miss out on life”). In addition, there were three images that (whilst coded within these themes) could have represented a separate category of loss/bereavement.

### Failure

**Figure 2:**
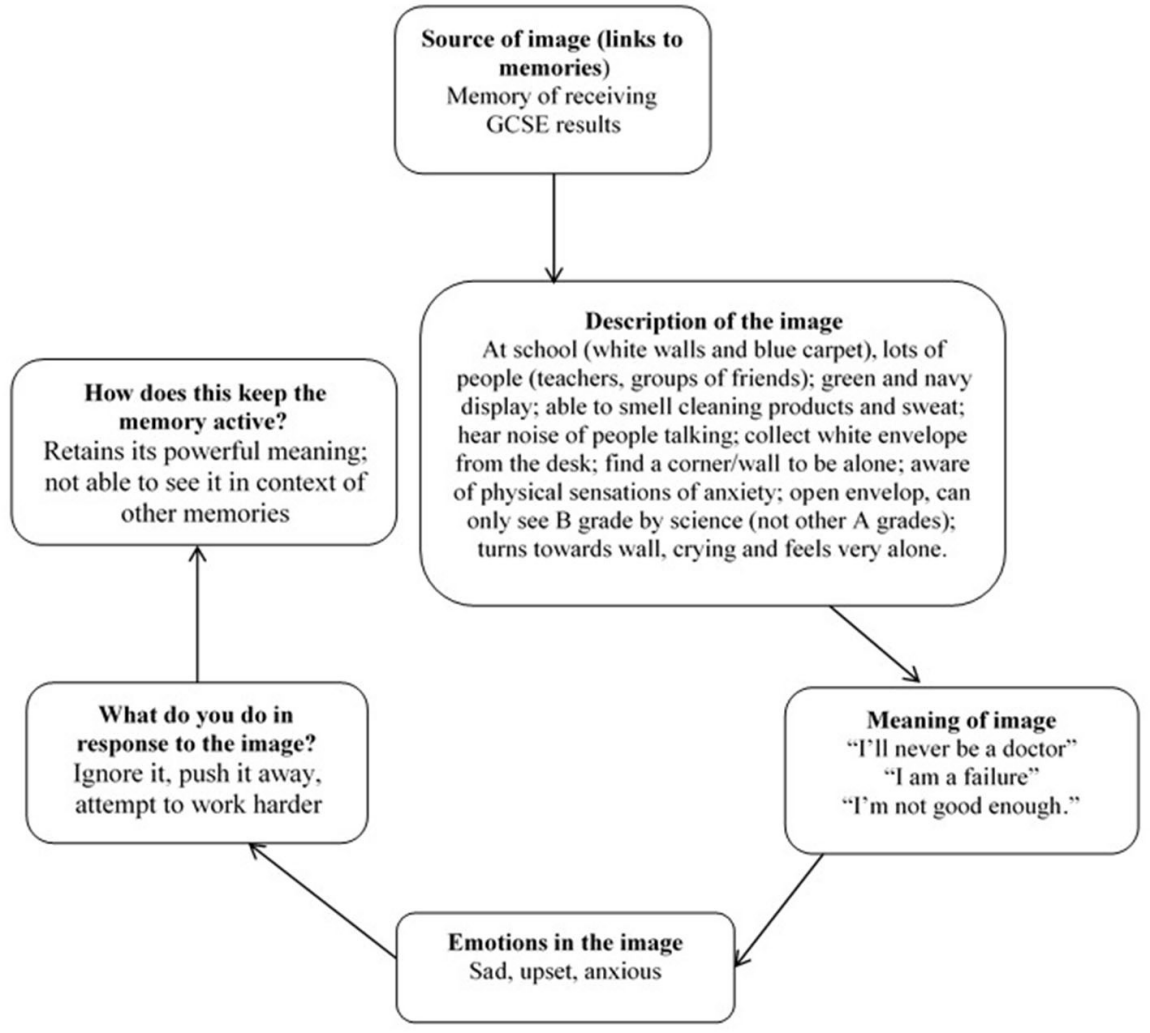
Example formulation of a negative non-traumatic image. The formulation is collaboratively completed in the IR session, during step 1.

Eight participants identified images of failure, most often failing an exam. For example, one participant (age 16, female, Asian/Asian British) described getting a B grade in in her science GCSE. She described science as her passion and wanting to become a General Practitioner. This image was having a significantly negative impact on her mood, motivation, and behavior. She spoke about attempting to revise for multiple hours after school and often instead staring blankly into space. During IR, she described the image in great detail (with the key moment being standing crying against a wall, staring at her results sheet). Key cognitions around this image were “I'm a failure” and “I'm not good enough.” She reported cognitively avoiding this image and needing to study at every opportunity to ensure it did not repeat itself. Please see Figure 2 for the formulation. She was able to clearly generate a compassionate other to intervene in the image. In this case, she chose her mother who saw her crying, approached and hugged her, and warmly assured her she has other options available and would get support from school to understand her next steps. Following rescripting, she spoke about the image no longer being “zoomed in” on the B grade and that she could now see it in context. She spoke about being more able to take a compassionate perspective toward herself, and to approach her academic work alongside her other values. Her depression scores reduced (MFQ score: Pre = 35; Post = 12; Follow-up = 4).

### Interpersonal Adversity

Fifteen participants identified images of interpersonal adversity, most commonly incidents of bullying, and rejection. For example, one young person (age 16, female, White British) spoke about being bullied and spoke about one particularly powerful intrusive image. This was of the group laughing at her in the classroom, and she left and closed the door (which shut loudly). When she returned, the group were impersonating her. She described feeling angry, being unsure of what to do or say. She described a sensation of rising heat. She was able to identify key cognitions as “I'm not as good as them” and “I'm so stupid to let them treat me like this.” She spoke about frequently ruminating about the event, e.g., “Why did this happen to me,” “What did I do wrong?.” She had since changed school and spoke about these experiences driving unhelpful behavior, such as avoiding friendships, not disclosing personal information and isolating herself. She acknowledged this was preventing her forming friendships at her new school. She generated her older cousin as a compassionate other, who intervened by checking she was OK and then responding to the group. The compassionate other removed her from the situation and then spent time thinking about what had happened, as well as highlighting her positive friendships and qualities. Following the intervention, she reported seeking out new friendships; no longer constantly questioning why the bullying had happened; significantly reducing her worry about future bullying; and standing up for herself more. Depression scores decreased (MFQ score: Pre = 42; Post = 18; Follow-up = 14).

### Family Conflict or Disruption

Thirteen participants identified images of family conflict or where family relationships had been disrupted. Examples include arguments between parents, parents leaving the family home or upsetting family events. One participant (age 17, male, White British) described having images of their mother being admitted to hospital. His mother was restrained and shouted some upsetting phrases at him. He described feeling angry, upset, and helpless. He described regularly having intrusive images about the event and “pushing” these out of his mind. Key cognitions included “how can she do this to me” and “it is my fault.” He had not previously spoken about this event and believes his mother does not remember her words. This was impacting on his relationship with his mother and his friends. He identified his father as a compassionate other who intervened, comforted him and took responsibility for the event. When providing feedback, he spoke about needing time after the session to process the event but now rarely experiencing intrusions. He spoke about the perspective-taking element (step 2) of the rescripting being “eye-opening,” as it had allowed him to acknowledge the impact the event had on him and that the event would have been difficult for anyone to cope with. Following IR, he was able to speak with his parents about what had happened and this had significantly improved their relationship. He also spoke about now being able to have their friends around to his house. Symptoms of depression decreased (MFQ score: Pre = 23; Post = 16; Follow-up = 6).

## Negative Consequences of IR

No adverse events were reported that were associated with the therapy. There were some adverse events reported during the RCT but these all had begun before the young person began therapy.

It seems likely that elevation in emotional arousal is important for emotional processing of the image. Almost all participants showed an increase in emotional arousal during step 1. Mood ratings were taken regularly throughout the session and, by the end of the session, ratings had almost always returned to near baseline levels. Participants frequently reported that they found the session emotionally challenging and it had a short-term negative impact (less than a day) on their mood but identified that, in the longer term, IR had been helpful for their mood and behavior. Finally, working clinically with distressing imagery can be emotionally challenging for the therapist. It is important to have supervision structures in place, including time to debrief on difficult sessions.

## Discussion

We have aimed to provide a comprehensive account of IR for adolescent depression. We have described the dearth of literature in young people, the developmental adaptations and stages in co-designing our protocol, three key themes of negative images with case examples, and that there were no reported negative consequences of IR. IR was a helpful tool to engage YP, explore and process negative recurrent memories, access key cognitions and decrease symptoms of depression.

No YP struggled to generate and manipulate a negative image. Negative images were accessible and frequent. This is important as the participants were selected for symptoms of depression, not for intrusive images. A trusting relationship is clearly important in allowing YP to engage with and manipulate imagery. There were concerns (before beginning) that the intervention might not be long enough to establish this relationship, this was not found to be the case. Session 1, including careful explanation and preparation work, is likely to have been important in forming this relationship. One advantage of IR is remaining flexible to what arises in step 1, as there were occasions where an unexpected, key cognition emerged. For example, for an image of being hit over the head by a peer, the key cognition was identified as “being weak” rather than the “world is dangerous” as first appeared.

The themes identified within the images (*failure, interpersonal adversity, family conflict, or disruption)* were consistent with the broader literature. For example, a qualitative study exploring what factors adolescents believe led to their depression (27), identified one key theme (of three) as being “depression as a result of rejection, victimization, and stress.” Another qualitative study identified five themes concerning the experience of adolescent depression (28), including “impact on education”; “isolation and cutting off from the world”; and “anger and violence toward self and others.” Research that could map the relationships between the content of intrusive images and the subjective experience of depression, as well as how this may vary across different cultures, would be valuable.

There were no examples of the young person being unable to identify a compassionate other. Most participants used a parent rather than an older self for this role. One therapeutic dilemma is whether to allow the compassionate other to prevent the event from taking place (rather than to intervene after the event). This is usually permitted in adult work. Whilst we did not give explicit instructions/guidelines about what the compassionate other could and couldn't do, none of the participants prevented the event taking place. In our view, it was helpful for the compassionate other to respond to the event (and for the participant to experience another perspective) rather than prevent the event from taking place. Further research could explore the relative benefits of preventing vs. intervening in the event.

Imagery rescripting appears to be an appropriate, acceptable and helpful intervention for YP with depression. A fully-powered trial could further test this approach and establish efficacy when delivered by non-specialist practitioners (to improve access). Although highly manualized and designed for non-specialist practitioners, the therapy was delivered by a clinical psychologist. Furthermore, whilst clinical judgement and feedback from YP (by questionnaires and qualitative interviews) suggests that IR was a key active ingredient in reducing depression, it would be helpful to establish the relative effects of the treatment components. Understanding developmental influences through experimental and/or longitudinal studies on the mechanisms of IR are important to translate effects to different age groups.

## Data Availability Statement

Data for this study are available in Mendeley Data. Pile (14), IMAGINE trial data, Mendeley Data V2. doi: 10.17632/7w3fwx7y2y.2.

## Ethics Statement

The studies involving human participants were reviewed and approved by Psychiatry, Nursing and Midwifery Research Ethics Committee at Kings College London (ref: HR-16/17-3548). The patients/participants provided their written informed consent to participate in this study.

## Author Contributions

VP: conceptualization, methodology, investigation, resources, writing—original draft, writing—review and editing, visualization, supervision, project administration, and funding acquisition. PS: conceptualization, methodology, writing—review and editing, and supervision. JL: conceptualization, methodology, resources, writing—review and editing, supervision, and project administration. All authors contributed to the article and approved the submitted version.

## Conflict of Interest

The authors declare that the research was conducted in the absence of any commercial or financial relationships that could be construed as a potential conflict of interest.

## Publisher's Note

All claims expressed in this article are solely those of the authors and do not necessarily represent those of their affiliated organizations, or those of the publisher, the editors and the reviewers. Any product that may be evaluated in this article, or claim that may be made by its manufacturer, is not guaranteed or endorsed by the publisher.
